# Pentadecanoic Acid (C15:0) at Naturally Occurring Circulating Concentrations Has Selective Anticancer Activities Including Targeting B-Cell Lymphomas with CCND3 Oncogenic Alterations

**DOI:** 10.3390/nu17193082

**Published:** 2025-09-28

**Authors:** Stephanie Venn-Watson

**Affiliations:** 1 Epitracker, Inc., San Diego, CA 92106, USA; stephanie@epitracker.com; 2 Seraphina Therapeutics, Inc., San Diego, CA 92106, USA

**Keywords:** C15:0, pentadecanoic acid, cancer, B-cell lymphoma, CCND3

## Abstract

**Background/Objectives:** While pentadecanoic acid (C15:0), present in whole dairy fat, has broad anticancer activities at high concentrations, the presence of C15:0 anticancer activities at naturally occurring circulating concentrations is less clear. **Methods:** Using an independent service to run the Eurofins OncoPanel^TM^ Cell Proliferation Assay, C15:0 was screened for dose-dependent antiproliferation activities against 94 human cancer cell lines at 10 concentrations ranging between 1.5 nM and 50 µM. Oncogenic alterations were compared between cell lines in which C15:0 did or did not have antiproliferation activities. **Results:** C15:0 had dose-dependent antiproliferation activities (EC50 ≤ 50 µM) among 13 (13.8%) cancer cell lines, most of which were non-Hodgkin B-cell lymphomas (n = 8, 61.5% of C15:0-responsive cell lines), but also included liver (n = 2, 15.4%), breast (n = 2, 15.4%), and lung (n = 1, 7.7%) cancers. C15:0 had robust antiproliferation activities (EC_50_, IC_50_ and GI_50_ ≤ 50 µM) in four cell lines, all of which were non-Hodgkin B-cell lymphomas. When comparing oncogenic alterations among C15:0-responsive versus non-responsive cancer cell lines (n = 79 with available data on DepMap), 4 of 18 (22%) C15:0-responsive cell lines had a CCND3 mutation compared to 1 of 61 (1.6%) non-responsive cell lines (*p* = 0.007, OR = 17.1, 95% CI 1.8–165). Three of four (75%) of the most C15:0-responsive B-cell lymphomas had the CCND3 alteration (*p* = 0.0004, OR = 180, 95% CI 8.9–3632). **Conclusions:** C15:0 has selective dose-dependent anticancer activities at naturally occurring concentrations. The potential use of C15:0 against cancers with CCND3 genetic alterations warrants further exploration. Further, there is a need to better understand the potential role of nutritional C15:0 deficiencies and CCND3 alterations on the observed rise in certain types of cancers, especially among young adults.

## 1. Introduction

Cancer remains a leading cause of mortality for adults aged 45 years or older [1]. Concerningly, the incidence of specific types of cancers, including colorectal, pancreatic, breast, kidney, and uterine cancer is on the rise among younger adults, especially in high-income countries [2]. The rise of specific cancers among young adults is coinciding with increases in the incidence of early-onset type 2 diabetes, atherosclerotic cardiovascular disease, and dementia [3,4,5]. While there are epidemiological links among these conditions, a clear underlying cause of the increase in cancer among younger adults is unclear [6,7,8,9].

Pentadecanoic acid (C15:0) is an odd-chain saturated fatty acid, primarily present in trace levels in dairy fat, that has emerged as an essential fatty acid and geroprotector [10,11,12,13,14,15]. C15:0 is an AMPK, AKT, and PPARɑ/δ activator and mTOR, JAK-STAT, and HDAC-6 inhibitor with optimal dose-dependent anti-inflammatory, antifibrotic, and antioxidant activities at concentrations ranging between 6.7 and 50 µM, which match that of those naturally occurring in human populations [15,16,17,18,19,20,21,22]. These pleiotropic mechanisms, paired with positive cell-based studies, in vivo efficacy studies, and clinical trials, support C15:0’s causative role in the observed lower risks of cardiovascular diseases, type 2 diabetes, and metabolic dysfunction-associated steatotic liver disease (MASLD) observed across diverse human populations [10,15,21,22,23,24,25,26,27,28,29,30,31].

People with higher C15:0 concentrations also have a lower risk of having certain types of cancers, including colorectal, breast, liver, pharyngolaryngeal, and bladder cancer [32,33,34,35,36,37]. Beyond association, C15:0 at higher doses (125 to 2000 µM) has the most effective and broad dose-dependent antiproliferation activities against human cancer cell lines compared to other fatty acids (including C5:0, C7:0, C9:0, and C11:0), including breast, pancreatic, lung, and liver cancers [20]. These anticancer activities have been attributed to C15:0’s role as an HDAC6, JAK-STAT and mTOR inhibitor [18,19,20]. Aligned with these studies, human cell-based phenotypic profiling showed that C15:0 at 50 µM, but not at lower concentrations, mimicked gemcitabine and paclitaxel, two leading anticancer drugs [38]. Further, in vivo treatment with gemcitabine and C15:0 as a prodrug effectively slowed and reduced tumor size in a rodent model with breast cancer better than gemcitabine alone [39]. Combined, these studies support that C15:0 has anticancer activities that are clinically relevant at higher concentrations (≥50 µM).

Population-wide circulating C15:0 concentrations are decreasing, which have been driven by lowered intake of whole dairy fat since the 1980s [40]. As a result, it is estimated that as many as 1 in 3 people globally may have nutritional C15:0 deficiencies that are contributing to the rise in type 2 diabetes, cardiovascular disease, and MASLD, especially in younger adults [13,41]. Given parallel increases in certain types of early-onset cancers, we sought to understand C15:0’s direct anticancer role as an essential fatty acid at naturally occurring human circulating C15:0 concentrations (≤50 µM). Here, pure C15:0 was tested for dose-dependent antiproliferative activities against 94 different human cancer cell lines, including breast (n = 17), liver (n = 6), lung (n = 27), and pancreatic (n = 13) cancers, as well as lymphomas (n = 31), at concentrations ranging from 1.5 nM to 50 µM.

Our studies show that C15:0 at concentrations consistent with naturally circulating levels in humans had the most robust dose-dependent antiproliferative activities against four human cancer cell lines, all of which were non-Hodgkin B-cell lymphomas. C15:0 showed a significant affinity for targeting B-cell lymphomas with CCND3 oncogenic alterations, which are more aggressive cancers [42]. Further, this study showed promise of C15:0 as a broader anticancer compound, including additional B cell lymphomas and breast, liver, lung, and pancreatic cancer cell lines at the higher end of naturally occurring circulating C15:0 concentrations (increasing from 16 to 50 µM). The potential use of C15:0 as a therapeutic against B-cell lymphomas and other cancers with CCND3 genetic alterations, known to be more aggressive cancers, warrants further exploration. Further, there is a need to better understand the potential role of nutritional C15:0 deficiencies, caused by population wide decreases in whole milkfat intake, on the observed rise in certain types of cancers, especially among young adults.

## 2. Materials and Methods

The OncoPanel™ by Eurofins (Eurofins Discovery Services, St. Charles, MO, USA) was conducted by Eurofins to measure the proliferation response of 97 cancer cell lines to 99% pure free fatty acid C15:0 (pentadecanoic acid, W433400, Sigma Aldrich, St. Louis, MO, USA) treatment through high-content fluorescence imaging or bioluminescence. Detailed methodology for the OncoPanel™ by Eurofins has been previously described [43]. Key methods most relevant to the current study are provided below.

### 2.1. Cell Proliferation Assay

Cells were grown in RPMI1640, 10%FBS, 2 mM L-alanyl-L-glutamine, 1 mM Na pyruvate, or a special medium. Cells were seeded into 384-well plates and incubated in a humidified atmosphere of 5% CO_2_ at 37 °C. C15:0 was added the day following cell seeding. At the same time, a time zero untreated cell plate was generated. After a 3-day incubation period, cells were fixed and stained to allow fluorescence imaging of nuclei.

Compounds were serially diluted in half-log steps from the highest test concentration specified in the above table and assayed over 10 concentrations (1.5 nM to 50 µM) with a maximum assay concentration of 0.2% DMSO. Secondary cell proliferation assays were performed among selected cancer cell lines, increasing the upper concentration to 100 µM. Automated fluorescence microscopy was carried out using a Molecular Devices ImageXpress Micro XL high-content imager (Molecular Devices, San Jose, CA, USA), and images were collected with a 4X objective. 16-bit TIFF images were acquired and analyzed with MetaXpress 5.1.0.41 software.

### 2.2. Data Analysis

Cell proliferation was measured by the fluorescence intensity of an incorporated nuclear dye. The output is referred to as the relative cell count, where the measured nuclear intensity is transformed to percent of control (POC) using the following formula: POC = *IxI*0 × 100. Where *Ix* is the nuclear intensity at concentration *x*, and *I*0 is the average nuclear intensity of the untreated vehicle wells. Cellular response parameters were calculated using nonlinear regression to a sigmoidal single-site dose response model. Time zero non-treated plates were used to determine the number of doublings during the assay period.

Cell count IC_50_ is the test compound concentration at 50% of maximal possible response. EC_50_ is the test compound concentration at the curve inflection point or half the effective response (parameter C of the fitted curve solution). GI_50_ is the concentration needed to reduce the observed growth by half (midway between the curve maximum and the time zero value). Curve-fitting, calculations, and report generation were performed using a custom data reduction engine and MathIQ-based software (AIM).

Cancer cell lines included in this study were evaluated for oncogenic alterations, as reported by DepMap [44]. Given two common oncogenic alterations that were identified among the four cell lines that had the most robust antiproliferative activities (defined as having EC_50_, IC_50_ and GI_50_ < 50 µM), significant differences in the prevalence of these two alterations (TP53 and CCDN3) were statistically compared among human cancer cell lines in which C15:0 did or did not have antiproliferation activities (defined as having an EC_50_ ≤ 50 µM or > 50 µM, respectively) using a two-way Chi-square test. Significance was defined as a *p*-value ≤ 0.05.

## 3. Results

### 3.1. C15:0 Had Selective Inhibitory Activities Against Specific Human Cancer Cell Types, Especially B-Cell Lymphomas

**Figure 1:**
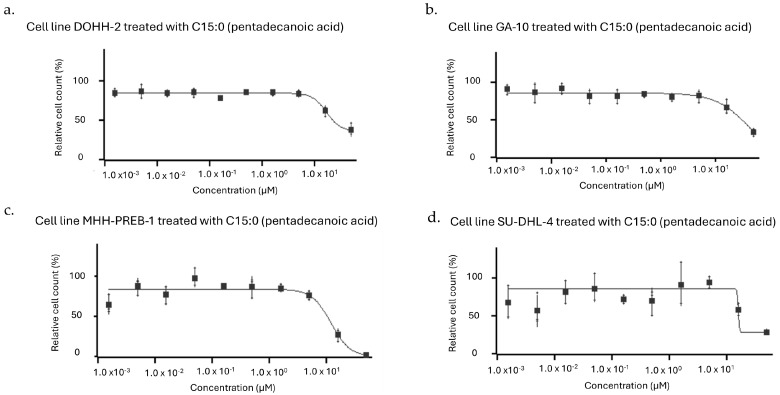
Dose–response curves (1.5 nm to 50 µM) of human cancer cell lines (**a**) DOHH-2, (**b**) GA-10, (**c**) MHH-PREB-1, and (**d**) SU-DHL-4 in which C15:0 had the most robust inhibitory activities (defined as EC_50_, IC_50_, and GI_50_ ≤ 50 µM). All of these cancer cell types were non-Hodgkin B-cell lymphomas.

Of the 97 human cancer cell lines included in the Eurofins OncoPanel, C15:0 antiproliferative data up to 50 µM were provided for 95 cell lines. One of these cell lines (Hs 229.T), while initially believed to be a lung adenocarcinoma, has since been shown to be a fibroblast cell line; as such, data from this cell line were excluded from the study [44]. C15:0 had inhibitory activities on 13 (13.8%) of the included 94 cell lines, in which the EC_50_ was ≤50 µM (Table 1). Among these 13 cancer cell lines, the C15:0 EC_50_ ranged from 6 to 47 µM. The majority (n = 8, 61.5%) of human cancer cell lines inhibited by C15:0 were non-Hodgkin B-cell lymphomas, but C15:0 also had antiproliferation activities against following cancer types: hepatocellular (n = 2, 15.4%), breast (n = 2, 15.4%), and lung (n = 1, 7.7%) cancers. Only four cancer cell lines had EC_50_, IC_50_ and GI_50_ ≤ 50 µM: DOHH-2, GA-10, MHH-PREB-1, and SU-DHL-4 (Figure 1). All of these are non-Hodgkin B cell lymphomas.

A summary of the 81 (86.2%) human cancer cell lines in which C15:0 did not have EC_50_, IC_50_, or GI_50_ ≤ 50 µM is provided in the Supplementary Data (Table S1). These included 25 (92.6%) of 26 lung cancer cell lines, 15 (88%) of 17 breast cancer cell lines, 13 of 13 (100%) of pancreatic cancer cell lines, 5 (71.4%) of 7 liver cancer cell lines, and 23 of 31 (74.2%) of lymphoma cells.

### 3.2. Lymphomas and Liver Cancers Had the Highest Prevalence of C15:0-Responsive Cell Lines

**Figure 2:**
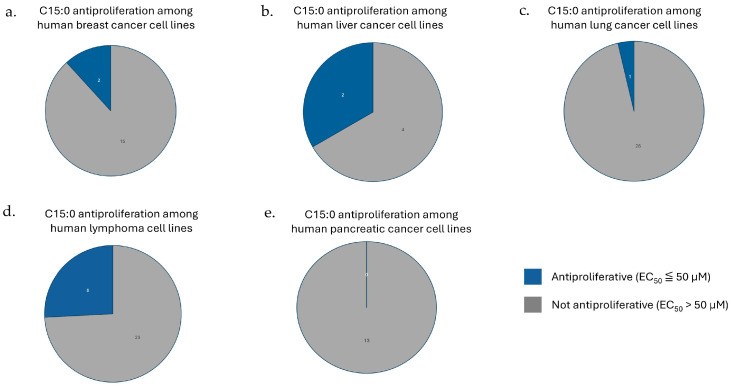
Pie graphs representing numbers and relative percentages of tested human cancer cell lines, by cancer type, in which C15:0 had EC50 ≤ or >50 µM for (**a**) breast cancers, (**b**) liver and bile duct cancers, (**c**) lung cancers, (**d**), lymphomas, and (**e**) pancreatic cancers.

The percentage of human cancer cell lines, by general cancer type, that had or did not have C15:0 antiproliferation activities (EC_50_ ≤ or >50 µM) is summarized in Figure 2. The cancer types that had the highest percentage of C15:0-responsive cell lines were liver and bile duct cancers (2 of 6, 33.3%) and lymphomas (8 of 31, 25.8%).

### 3.3. Human Cancer Cell Lines Were More Likely to Be Responsive to C15:0 if They Had CCND3 Oncogenic Alterations

To better understand why C15:0 antiproliferation activities are selective at lower concentrations, oncogenic alterations, as reported by DepMap, were compared among human cancer cell lines that were responsive versus non-responsive to C15:0 [45]. Of the 94 cell lines, oncogenic alteration data were available for 79 (84%) (Table S2). The four cell lines that had the most robust antiproliferative activities (DOHH-2, GA-10, MHH-PREB-1, and SU-DHL-4) shared two oncogenic alterations: TP53 loss-of-function (3 of 4 cell lines) and CCND3 gain-of-function (3 of 4 cell lines) (Table 2).

When comparing TP53 loss-of-function oncogenic alterations among C15:0-responsive versus non-responsive cancer cell lines (n = 18 C15:0-responsive cell lines, n = 61 non-C15:0-responsive cell lines), 14 (78%) C15:0-responsive cell lines had a TP53 oncogenic alteration compared to 38 (62%) non-responsive cell lines (*p* = 0.11, 95% CI 0.6–7.2). Three of four (75%) of the most robust C15:0-responsive cancer cell lines (GA-10, MHH-PREB-1 and SU-DHL-4) had the TP53 alteration, which was not significantly higher than non-C15:0-responsive cell lines (*p* = 0.31, 95% CI 0.2–18.5).

When comparing CCND3 gain-of-function oncogenic alterations among C15:0-responsive versus non-responsive cancer cell lines (n = 18 C15:0-responsive cell lines, n = 61 non-C15:0-responsive cell lines), 4 (22%) C15:0-responsive cell lines had a CCND3 oncogenic alteration compared to 1 (1.6%) non-responsive cell line (*p* = 0.007, OR = 17.1, 95% CI 1.8–165). Three of four (75%) of the most robust C15:0-responsive cancer cell lines (DOHH-2, GA-10 and MHH-PREB-1) had the CCND3 alteration, which was significantly higher than non-C15:0-responsive cell lines (*p* = 0.0004, OR = 180, 95% CI 8.9–3632).

### 3.4. C15:0 Has Inhibitory Activities Among Additional Cancer Cell Lines as C15:0 Treatment Increased from 16 to 50 µM

**Figure 3:**
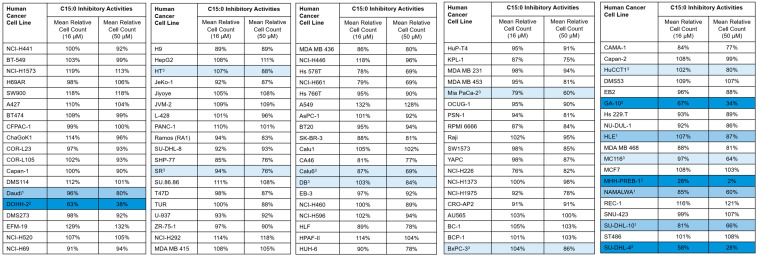
Human cancer cell types that had at least a 15% decrease in mean relative cell count when C15:0 treatment concentrations increased from 16 to 50 µM. Mid-blue: ^1^ EC_50_ ≤ 50 µM; Dark blue: ^2^ EC_50_, IC_50_ and GI_50_ ≤ 50 µM; Light blue: ^3^ EC_50_, IC_50_ and GI_50_ > 50 µM.

It was observed that, while some human cancer cell lines did not have C15:0 EC_50_, IC_50_, or GI_50_ concentrations ≤ 50 µM, eight additional cell lines had a meaningful decrease (defined as at least a 15% decrease in relative percent cell count) when comparing the two highest concentrations tested, from 16 to 50 µM (Figure 3). This included the following cancer types: lung (n = 1), pancreatic (n = 2), liver (n = 1), B cell lymphomas (n = 4).

**Figure 4:**
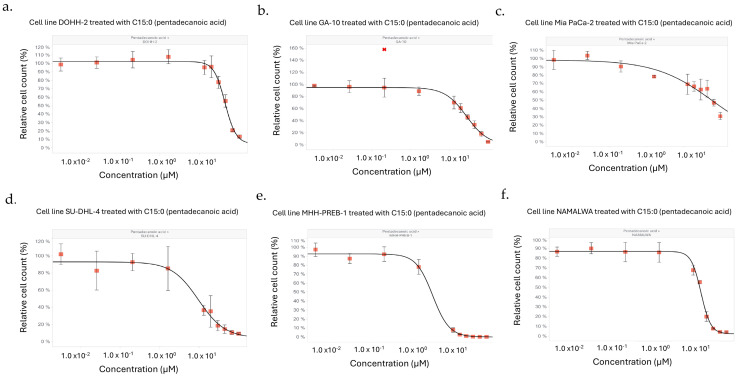
Dose–response curves (up to 100 µM) of six human cancer cell types, specifically (**a**) DOHH-2, (**b**) GA-10, (**c**) Mia PaCa-2, (**d**) SU-DHL-4, (**e**) MHH-PREB-1, and (**f**) NAMALWA, in which C15:0 treatment at 50 µM resulted in relative cell counts ≤ 60%. Five of these cancer cell types were B cell lymphomas and one (Mia PaCa-2) was a pancreatic cancer cell line.

As such, C15:0 inhibitory activities were tested at higher concentrations (3.2 nM to 100 µM) for the four cancer cell lines that had EC_50_, IC_50_ and GI_50_ ≤ 50 µM, along with two additional cell lines in which the mean relative cell count at 50 µM decreased to ≤60% (NAMALWA and Mia Pa-Ca-2) (Figure 4). C15:0 at these higher doses had significant, dose-dependent antiproliferative effects (EC_50_, IC_50_ and GI_50_ ≤ 50 µM) across all six cancer cell lines, including a pancreatic cancer cell line (Mia Pa-Ca-2) (Table 3).

## 4. Discussion

At concentrations reasonably and naturally present in humans (≤50 µM), C15:0 had significant and selective dose-dependent antiproliferation activities against four specific non-Hodgkin B-cell lymphoma cell lines, with IC_50_ ranging from 12 to 38 µM. This C15:0-responsive selectivity appears to be attributable, at least in part, to C15:0 targeting cancers with CCND3 oncogenic gain-of-function alterations. This is the first report of C15:0’s selective targeting of cancers at naturally occurring concentrations, which are attributable at least in part to B-cell lymphomas with CCND3 oncogenic alterations.

This finding complements the existing literature on broader antiproliferative and anticancer activities of C15:0 at higher concentrations. Do et al. previously demonstrated that C15:0 decreased colony formation and induced apoptosis in HepG2 (IC_50_ 178 ± 8 µM) and Huh7 (IC_50_ 120 ± 9 µM) hepatocellular carcinoma cell lines, especially at concentrations of 200 and 300 µM [46]. C15:0 cancer-specific apoptotic activities were based on increases in the sub-G1 population and increased C-Caspase 3/Caspase 3 and C-PARP/PARP ratios. Similarly, Ediriweera et al. showed that C15:0 had broad, dose-dependent antiproliferative effects at higher concentrations across eight different human cancer cell lines, including breast, pancreatic, lung, and liver cancers (mean IC_50_ ranging from 130 to 260 µM) [20]. These anticancer activities were the strongest compared to other saturated fatty acids and were attributed to the demonstrated role as C15:0 an HDAC inhibitor at similar concentrations (IC_50_ of 200 µM). This same team also showed that C15:0 exerted cytotoxicity by inducing apoptosis in MCF-7 and the more aggressive MCF-7/SC human breast cancer cell lines, especially at 200 µM [19]. Further, C15:0 inhibited migration and invasion of MCF-7/SC in wound healing and trans-well invasion assays, supporting that C15:0 could inhibit the invasiveness of migration of at least certain types of cancers. These effects were attributed to demonstrated C15:0 suppression of JAK2/STAT3 signaling in MCF-7/SC. In yet another study by this team, To et al. showed that C15:0 reversed tamoxifen resistance in MCF-7/SC breast cancer cells, in part due to C15:0’s suppression of mTOR, especially at 100 µM [18]. These earlier studies consistently demonstrated C15:0’s antiproliferative, apoptotic, and antimigratory activities against a broad array of human cancer cell lines at higher doses (100 to 300 µM) due to pleiotropic mechanisms of action, including HDAC6, JAK2/STAT3, and mTOR inhibition. There is a need to understand the mechanisms of action of C15:0 at lower concentrations (12 to 38 µM) to explain its specificity against non-Hodgkin B cell lymphomas, especially those with CCND3 oncogenic alterations.

Beyond these cell-based studies, C15:0 increases mean survival time and decreases tumor size in animal models. Isoda et al. injected a variety of saturated fatty acids, ranging from C6:0 to C24:0, in mice with sarcomas [47]. Consistent with cell-based studies, C15:0 had the greatest anticancer effects compared to other fatty acids. This study showed that daily C15:0 injected at doses of 1.25 mg or 5 mg resulted in a dose–response increase in survival days by 28.8 ± 6.9 and 42.2 ± 15.7, respectively, compared to non-treated controls. These outcomes translated to a 162% and 284% increased lifespan, respectively. In a separate study, Li et al. showed that C15:0 combined with gemcitabine as a prodrug (called “GZ”) substantially lowered the IC_50_ of gemcitabine alone against eight of nine human cancer cell lines [39]. Further, in mice implanted with 4T1 mouse breast cancer cells, GZ at 5 mg/kg and 10 mg/kg resulted in significantly decreased tumor volume and tumor weight compared to gemcitabine alone. These anticancer effects did not impair healthy tissues, including the heart, liver or kidney. These studies confirm that C15:0, as an injectable, has direct and safe in vivo anticancer effects in animals.

In the current study, C15:0 had selective antiproliferative activities at naturally occurring concentrations against specific types of cancers, especially non-Hodgkin B-cell lymphomas with CCND3 alterations. D-type cyclins are proteins that regulate cell cycle progression from the G1 to S stage, especially in cancer cells [48]. These cyclins include three protein-coding genes: CCND1, CCND2, and CCND3. The CCND3 gene is considered a strongly selective and druggable structure by bioactive ligand compounds, and it is most highly expressed in bone marrow and lymphoid tissues [49,50,51]. As such, compounds that down-regulate CCND3 have been proposed as targeted therapeutic candidates for lymphomas and osteosarcomas that have this oncogenic alteration [52,53,54]. Given the current study’s results and C15:0 safety as a nutrient, dosing C15:0 toward the higher end of naturally occurring concentrations should be explored as a potential therapeutic option for individuals with CCND3-related cancers.

In our study, two of four C15:0-responsive cancer cell lines that had the CCND3 oncogenic alteration were diffuse large B-cell lymphomas (DLBCL). DLBCL represents 30 to 40% of newly diagnosed lymphomas and is associated with decreased survival [52]. In a study including 2059 patients with DLBCL, 5.5% had CCND3 gene mutations, demonstrating that there is a sizable population with this oncogenic alteration [52]. In addition to DLBCL, our current study identified a C15:0-responsive CCND3 cancer cell line from a patient with Burkitt lymphoma. CCND3 mutations are equally frequent among adult and pediatric patients with Burkitt lymphoma, which are present in greater than 20% of patients [55]. Additionally, CCND3 has been characterized as “indispensable” for B-cell proliferation in acute lymphoblastic leukemia due to its surprisingly independent role in inhibiting apoptosis of neoplastic B cells and fostering resistance to anticancer therapeutics, such as palbociclib [53]. These studies support screening patients with B-cell lymphomas and acute lymphoblastic leukemias for CCND3 genetic alterations, which appear to be more responsive to lower concentration C15:0 as a candidate anticancer compound.

CCND3 mutations are also relatively common in osteosarcomas, representing 14.8% of patients in one study [56]. Compared to adults with osteosarcoma, pediatric and adolescent patients are significantly more likely to have osteosarcomas with CCND3 alterations. Further, similar to lymphomas, CCND3 mutations are associated with more aggressive cancer [57]. As such, identifying patients with this mutation has been proposed to help target optimal treatment options [54]. More studies are needed to further evaluate C15:0 as a potential targeted treatment for patients, especially younger patients, with CCND3-related osteosarcomas.

Beyond lymphomas and osteosarcomas, CCND3 mutations have been identified in additional malignant cancers, including breast, bladder, colorectal, hepatocellular, non-small cell lung cancers and malignant gliomas [58,59,60,61,62]. Epidemiological studies have shown that people with higher circulating C15:0 concentrations are less likely to have breast, bladder, colorectal, and hepatocellular cancers [32,33,34,35,36,37]. As such, there is a need to better understand if people with low C15:0 concentrations may be more likely to have these types of cancers due to CCND3 mutations. Understanding that population wide C15:0 concentrations are declining due to decreased consumption of whole fat dairy products, this line of investigation is gaining increasing importance [40].

At the higher range of normal circulating concentrations (increasing from 16 to 50 µM), C15:0 had antiproliferative activities against additional B-cell lymphomas, as well as specific breast, liver, lung, and pancreatic human cancer cell lines. Further, aside from one B-cell lymphoma case, these cancer cell lines did not have the CCND3 mutation. When exploring C15:0 antiproliferative activities of some of these cell lines at concentrations higher than what is naturally occurring (50 to 100 µM), all had significant anticancer activities, including a pancreatic adenocarcinoma cell line (IC_50_ 63 µM). The more generalized antiproliferative activities of C15:0 at higher concentrations is consistent with our prior report of C15:0 cell-based phenotypic profiling that closely matched gemcitabine and paclitaxel [38]. Further, higher concentrations (125 to 2000 µM) of C15:0 have shown universal anticancer activities against all breast, pancreatic, lung, and liver cancer cell lines tested while maintaining safety in non-neoplastic cells [20].

Limitations of this study include use of immortalized human cancer cell lines, which can change over time and not reflect the same outcomes as using primary cancer cells directly from newer patients. In an earlier study, we showed that C15:0 had direct, dose-dependent antiproliferative effects on primary human B cells in a cell system mimicking chronic inflammation and hematologic oncology [38]. This cell-based phenotypic profiling study, which used only primary human cells, also demonstrated a significant match of C15:0 activities at 50 µM with that of gemcitabine and paclitaxel. While these primary cell systems did not use primary cancer cells, this study supports that C15:0 has anticancer activities in primary cells as well as immortalized human cancer cell lines. Future studies, however, should include primary cancer cells.

An additional limitation of the current study, given the thesis that nutritional C15:0 deficiencies may be potential drivers for the increased prevalence of certain types of cancers in some populations, is the need to confirm that C15:0 anticancer activities remain present upon ingestion of either (1) pure, free fatty acid C15:0 or (2) C15:0 from the diet, which is in a triglycerol form. Triacylglycerol C15:0 in foods, including dairy fat, is broken down by digestive enzymes into free fatty acid C15:0, which can be absorbed [63]. Once absorbed, C15:0 is incorporated into many different lipid species, such as phospholipids, lysophospholipids, and cholesterol esters. Due to the complexity of C15:0 across many different lipids, studies typically measure circulating and tissue C15:0 levels as total C15:0, which includes all C15:0 present in all lipid species. This method has reliably demonstrated through numerous studies the following: (1) ingestion of C15:0 as a free fatty acid or as C15:0 within foods reliably increases total C15:0 levels, and (2) ingestion of free fatty acid C15:0 results in in vivo benefits that were demonstrated in vitro. For example, we and others have shown that C15:0 decreases proliferation of fibroblasts in cell systems mimicking liver fibrosis and decreases proinflammatory cytokines (e.g., MCP-1 and TNF-α) in cell systems mimicking chronic inflammation [10]. Further, we have shown that providing daily oral, free fatty acid C15:0 for 12 weeks results in lowered liver fibrosis and lower MCP-1 and TNF-α in relevant animal models [10]. As another example, C15:0 decreases biomarkers of restenosis in human vascular cell systems and increases dietary intake of C15:0 from foods results in raised circulating C15:0 concentrations that are correlated with improved vascular function [15,26]. These studies support that C15:0 cell-based efficacy successfully predicts efficacy of orally administered C15:0, either as a free fatty acid or from the diet.

As further support, epidemiological studies have shown that people with higher C15:0 concentrations have a lower risk of having certain types of cancers, including colorectal, breast, liver, pharyngolaryngeal, and bladder cancer [32,33,34,35,36,37]. This includes B-cell lymphomas and other hematologic malignancies. Hori et al. showed that serum sphingomyelin C15:0 concentrations were significantly lower in people with B-cell lymphoma, myelodysplastic syndrome, and acute lymphatic leukemia/lymphoblastic lymphoma (ALL/BLL) compared to healthy controls; this was not the case for 14 out of 16 of the other fatty acids included in this study [64]. Due to the current study’s demonstrated anticancer activities of C15:0 at naturally occurring circulating levels in humans, these combined studies support that higher C15:0 concentrations may have a causative role in lowering the risk of certain types of cancers. Clinical trials, however, are needed to confirm that ingested C15:0 as a free fatty acid or within food, exerts anticancer activities.

## 5. Conclusions

In summary, the current study shows that C15:0’s anticancer role evolves from targeting specific cancers with CCND3 mutations to broad anticancer activities as C15:0 concentrations increase from naturally to non-naturally occurring concentrations. Further studies are needed to understand the role of optimizing naturally occurring C15:0 concentrations, especially given population wide declining C15:0 levels that are coinciding with increased specific types of cancers among younger people.

## 6. Patents

U.S. Patent Application No. 63/880986.

## Figures and Tables

**Table 1:** Human cancer cell lines in which pentadecanoic acid (C15:0) had significant antiproliferative activities ≤ 50 µM. EC_50_ = C15:0 concentration at the curve inflection point, or half the effective response. IC_50_ = C15:0 concentration at 50% of maximal possible response. GI_50_ = C15:0 concentration needed to reduce the observed growth by half (midway between the curve maximum and the time zero value).

Human Cancer Cell Line	Cancer Type	Dose-Dependent Antiproliferative Effect of C15:0 (1.5–50 µM)		
		Cell Count EC_50_ (µM)	Cell Count IC_50_ (µM)	Cell Count GI_50_ (µM)
Daudi	Lymphoma (Burkitt Lymphoma, Mature B-Cell Neoplasm)	34	>50	>50
DOHH-2	Lymphoma (Diffuse Large B-Cell Lymphoma, NOS, Mature B-Cell Neoplasm)	17	31	25
GA-10	Lymphoma (Burkitt Lymphoma, Mature B-Cell Neoplasm)	38	38	31
HLE	Hepatocellular Carcinoma	48	>50	>50
HLF	Hepatocellular Carcinoma	6.2	>50	>50
JeKo-1	Lymphoma (Mantle Cell Lymphoma, Mature B-Cell Neoplasm)	47	>50	>50
MHH-PREB-1	Lymphoma (Non-Hodgkin Lymphoma)	12	12	11
NAMALWA	Lymphoma (Burkitt Lymphoma, Mature B-Cell Neoplasm)	18	>50	>50
SHP-77	Lung Cancer (Small Cell Lung Cancer Lung Neuroendocrine Tumor)	17	>50	>50
SU-DHL-4	Lymphoma (Diffuse Large B-Cell Lymphoma, NOS, Mature B-Cell Neoplasm)	16	16	16
SU-DHL-10	Lymphoma (Diffuse Large B-Cell Lymphoma, NOS, Mature B-Cell Neoplasm)	18	>50	>50
T47D	Breast Cancer (Breast Invasive Carcinoma)	42	>50	>50
ZR-75-1	Breast Cancer (Breast Invasive Carcinoma)	39	>50	>50

**Table 2:** Presence or absence of TP53 and CCND3 oncogenic alterations in human cancer cell lines in which pentadecanoic acid (C15:0) had significant antiproliferative activities (EC50 ≤ 50 µM).

Cell Line	C15:0 Inhibition Activities (EC, IC and GI_50_ ≤ 50 µM)	General Cancer Type	TP53 Loss of Function	CCND3 Gain of Function
DOHH-2	Yes	Lymphoma	No	Yes
GA-10			Yes	Yes
MHH-PREB-1			Yes	Yes
SU-DHL-4			Yes	No
SU-DHL-10	No	Lymphoma	No	Yes
Daudi		Lymphoma	Yes	No
HT		Lymphoma	Yes	No
CALU6		Lung	No	No
Mia PaCa-2		Pancreatic	Yes	No
BxPC-3		Pancreatic	Yes	No
HuCCT1		Liver	Yes	No
HLE		Liver	Yes	No
MC116		Lymphoma	Yes	No
NAMALWA		Lymphoma	Yes	No
T47D		Breast	Yes	No
ZR-75-1		Breast	No	No
SHP-77		Lung	Yes	No
HLF		Liver	Yes	No

**Table 3:** Selected human cancer cell lines in which pentadecanoic acid (C15:0) had significant antiproliferative activities ≤ 100 µM. EC_50_ = C15:0 concentration at the curve inflection point, or half the effective response. IC_50_ = C15:0 concentration at 50% of maximal possible response. GI_50_ = C15:0 concentration needed to reduce the observed growth by half (midway between the curve maximum and the time zero value).

Human Cancer Cell Line	Cancer Type	Dose-Dependent Antiproliferative Effect of C15:0 (3.2 nM–100 µM)		
		Cell Count EC_50_ (µM)	Cell Count IC_50_ (µM)	Cell Count GI_50_ (µM)
DOHH-2	Lymphoma (Diffuse Large B-Cell Lymphoma, NOS, Mature B-Cell Neoplasm)	44	45	38
GA-10	Lymphoma (Burkitt Lymphoma, Mature B-Cell Neoplasm)	28	28	24
MHH-PREB-1	Lymphoma (Non-Hodgkin Lymphoma)	0.4	0.4	0.4
NAMALWA	Lymphoma (Burkitt Lymphoma, Mature B-Cell Neoplasm)	21	22	20
SU-DHL-4	Lymphoma (Diffuse Large B-Cell Lymphoma, NOS, Mature B-Cell Neoplasm)	9.7	10.4	6.0
Mia PaCa-2	Pancreatic (Pancreatic Adenocarcinoma)	62	63	41

## Data Availability

Data for this study are provided as Supplementary Information.

## Supplementary Materials

The following supporting information can be downloaded at https://www.mdpi.com/article/10.3390/nu17193082/s1. Table S1: Human Cancer Cell Lines in which C15:0 had no Dose-Dependent Inhibitory Activities at Concentrations < 50 µM; Table S2: Oncogenic Alterations (TP53 and CCND3) in Human Cancer Cell Lines by C15:0 Inhibition Activities; C15:0 Treated vs. Vehicle Data Tables.

## Abbreviations

The following abbreviations are used in this manuscript:

C15:0	Pentadecanoic acid
EC_50_	The test compound concentration at the curve inflection point or half the effective response
IC_50_	The test compound concentration at 50% of maximal possible response
GI_50_	The test compound concentration needed to reduce the observed growth by half (midway between the curve maximum and the time zero value)
CCND3	D-cyclin type gene
AMPK	AMP-activated protein kinase
AKT	Protein kinase B
PPARα/δ	Peroxisome proliferator-activated receptor alpha/delta
mTOR	Mechanistic target of rapamycin
JAK-STAT	Janus kinase-signal transducer and activator of transcription
HDAC-6	Histone deacetylase 6
MASLD	Metabolic dysfunction-associated steatotic liver disease
DLBCL	Diffuse large B-cell lymphoma
DMSO	Dimethyl sulfoxide
POC	Point of control
