# Supplementary material for: Pentadecanoic Acid (C15:0) at Naturally Occurring Circulating Concentrations Has Selective Anticancer Activities Including Targeting B-Cell Lymphomas with CCND3 Oncogenic Alterations
Source: Nutrients. 2025 Sep 28;17(19):3082. doi: 10.3390/nu17193082 (PMC12525904; doi:10.3390/nu17193082)
Supplement: Supplementary file 1 [file nutrients-17-03082-s001.zip › Venn-Watson et al. 2025 C15 vs. Vehicle Antiproliferation Data - SUPPLEMENTARY DATA.pdf]

Summary

Summary table for test compounds

Compound	Cell Line	Cell Count EC50 (microM)	Cell Count IC50 (microM)	Cell Count GI50 (microM)	Cell Count Activity Area
C15:0 (pentadecanoic acid)	NCI-H441	> 5.00E+01	> 5.00E+01	> 5.00E+01	0.28
C15:0 (pentadecanoic acid)	BT-549	> 5.00E+01	> 5.00E+01	> 5.00E+01	0.18
C15:0 (pentadecanoic acid)	NCI-H1573	> 5.00E+01	> 5.00E+01	> 5.00E+01	0.03
C15:0 (pentadecanoic acid)	H69AR	> 5.00E+01	> 5.00E+01	> 5.00E+01	0.09
C15:0 (pentadecanoic acid)	SW900	> 5.00E+01	> 5.00E+01	> 5.00E+01	0.16
C15:0 (pentadecanoic acid)	A427	> 5.00E+01	> 5.00E+01	> 5.00E+01	0.06
C15:0 (pentadecanoic acid)	BT474	> 5.00E+01	> 5.00E+01	> 5.00E+01	0.63
C15:0 (pentadecanoic acid)	CFPAC-1	> 5.00E+01	> 5.00E+01	> 5.00E+01	0.28
C15:0 (pentadecanoic acid)	ChaGoK1	> 5.00E+01	> 5.00E+01	> 5.00E+01	0.25
C15:0 (pentadecanoic acid)	COR-L23	> 5.00E+01	> 5.00E+01	> 5.00E+01	0.52
C15:0 (pentadecanoic acid)	COR-L105	> 5.00E+01	> 5.00E+01	> 5.00E+01	0.19
C15:0 (pentadecanoic acid)	Capan-1	> 5.00E+01	> 5.00E+01	> 5.00E+01	0.85
C15:0 (pentadecanoic acid)	DMS114	> 5.00E+01	> 5.00E+01	> 5.00E+01	0.12
C15:0 (pentadecanoic acid)	Daudi	3.38E+01	> 5.00E+01	> 5.00E+01	0.33
C15:0 (pentadecanoic acid)	DOHH-2	1.68E+01	3.08E+01	2.50E+01	0.83
C15:0 (pentadecanoic acid)	DMS273	> 5.00E+01	> 5.00E+01	> 5.00E+01	0.14
C15:0 (pentadecanoic acid)	EFM-19	> 5.00E+01	> 5.00E+01	> 5.00E+01	0.26
C15:0 (pentadecanoic acid)	SKMES1	> 5.00E+01	> 5.00E+01	> 5.00E+01	0.26
C15:0 (pentadecanoic acid)	NCI-H520	> 5.00E+01	> 5.00E+01	> 5.00E+01	0.43
C15:0 (pentadecanoic acid)	NCI-H69	> 5.00E+01	> 5.00E+01	> 5.00E+01	0.06
C15:0 (pentadecanoic acid)	H9	> 5.00E+01	> 5.00E+01	> 5.00E+01	0.80
C15:0 (pentadecanoic acid)	HepG2	> 5.00E+01	> 5.00E+01	> 5.00E+01	0.07
C15:0 (pentadecanoic acid)	HT	> 5.00E+01	> 5.00E+01	> 5.00E+01	0.26
C15:0 (pentadecanoic acid)	JeKo-1	4.70E+01	> 5.00E+01	> 5.00E+01	0.50
C15:0 (pentadecanoic acid)	Jiyoye	> 5.00E+01	> 5.00E+01	> 5.00E+01	0.69
C15:0 (pentadecanoic acid)	JVM-2	> 5.00E+01	> 5.00E+01	> 5.00E+01	0.47
C15:0 (pentadecanoic acid)	L-428	> 5.00E+01	> 5.00E+01	> 5.00E+01	0.36
C15:0 (pentadecanoic acid)	PANC-1	> 5.00E+01	> 5.00E+01	> 5.00E+01	0.44
C15:0 (pentadecanoic acid)	Ramos (RA 1)	> 5.00E+01	> 5.00E+01	> 5.00E+01	0.24
C15:0 (pentadecanoic acid)	SU-DHL-8	> 5.00E+01	> 5.00E+01	> 5.00E+01	0.54
C15:0 (pentadecanoic acid)	SHP-77	1.66E+01	> 5.00E+01	> 5.00E+01	0.57
C15:0 (pentadecanoic acid)	SR	> 5.00E+01	> 5.00E+01	> 5.00E+01	0.27
C15:0 (pentadecanoic acid)	SU.86.86	> 5.00E+01	> 5.00E+01	> 5.00E+01	0.23
C15:0 (pentadecanoic acid)	T47D	4.23E+01	> 5.00E+01	> 5.00E+01	0.25
C15:0 (pentadecanoic acid)	TUR	> 5.00E+01	> 5.00E+01	> 5.00E+01	0.23
C15:0 (pentadecanoic acid)	U-937	> 5.00E+01	> 5.00E+01	> 5.00E+01	0.42
C15:0 (pentadecanoic acid)	ZR-75-1	3.91E+01	> 5.00E+01	> 5.00E+01	0.48
C15:0 (pentadecanoic acid)	NCI-H292	> 5.00E+01	> 5.00E+01	> 5.00E+01	0.39
C15:0 (pentadecanoic acid)	MDA MB 415	> 5.00E+01	> 5.00E+01	> 5.00E+01	0.21
C15:0 (pentadecanoic acid)	MDA MB 436	> 5.00E+01	> 5.00E+01	> 5.00E+01	0.77
C15:0 (pentadecanoic acid)	NCI-H446	> 5.00E+01	> 5.00E+01	> 5.00E+01	0.17
C15:0 (pentadecanoic acid)	Hs 578T	> 5.00E+01	> 5.00E+01	> 5.00E+01	0.74
C15:0 (pentadecanoic acid)	NCI-H661	> 5.00E+01	> 5.00E+01	> 5.00E+01	0.67
C15:0 (pentadecanoic acid)	Hs 766T	> 5.00E+01	> 5.00E+01	> 5.00E+01	0.30
C15:0 (pentadecanoic acid)	A549	> 5.00E+01	> 5.00E+01	> 5.00E+01	0.72
C15:0 (pentadecanoic acid)	AsPC-1	> 5.00E+01	> 5.00E+01	> 5.00E+01	0.09
C15:0 (pentadecanoic acid)	BT20	> 5.00E+01	> 5.00E+01	> 5.00E+01	0.20
C15:0 (pentadecanoic acid)	SK-BR-3	> 5.00E+01	> 5.00E+01	> 5.00E+01	0.68
C15:0 (pentadecanoic acid)	Calu1	> 5.00E+01	> 5.00E+01	> 5.00E+01	0.42
C15:0 (pentadecanoic acid)	CA46	> 5.00E+01	> 5.00E+01	> 5.00E+01	1.07

C15:0 (pentadecanoic acid)	Calu6	> 5.00E+01	> 5.00E+01	> 5.00E+01	0.78
C15:0 (pentadecanoic acid)	DB	> 5.00E+01	> 5.00E+01	> 5.00E+01	0.32
C15:0 (pentadecanoic acid)	EB-3	> 5.00E+01	> 5.00E+01	> 5.00E+01	0.74
C15:0 (pentadecanoic acid)	NCI-H460	> 5.00E+01	> 5.00E+01	> 5.00E+01	0.42
C15:0 (pentadecanoic acid)	NCI-H596	> 5.00E+01	> 5.00E+01	> 5.00E+01	0.26
C15:0 (pentadecanoic acid)	HLF	6.21E+00	> 5.00E+01	> 5.00E+01	0.84
C15:0 (pentadecanoic acid)	HPAF-II	> 5.00E+01	> 5.00E+01	> 5.00E+01	0.11
C15:0 (pentadecanoic acid)	HUH-6 Clone 5	> 5.00E+01	> 5.00E+01	> 5.00E+01	1.98
C15:0 (pentadecanoic acid)	HuP-T4	> 5.00E+01	> 5.00E+01	> 5.00E+01	0.42
C15:0 (pentadecanoic acid)	KPL-1	> 5.00E+01	> 5.00E+01	> 5.00E+01	0.93
C15:0 (pentadecanoic acid)	MDA MB 231	> 5.00E+01	> 5.00E+01	> 5.00E+01	0.78
C15:0 (pentadecanoic acid)	MDA MB 453	> 5.00E+01	> 5.00E+01	> 5.00E+01	0.29
C15:0 (pentadecanoic acid)	Mia PaCa-2	> 5.00E+01	> 5.00E+01	> 5.00E+01	0.72
C15:0 (pentadecanoic acid)	OCUG-1	> 5.00E+01	> 5.00E+01	> 5.00E+01	0.59
C15:0 (pentadecanoic acid)	PSN-1	> 5.00E+01	> 5.00E+01	> 5.00E+01	0.44
C15:0 (pentadecanoic acid)	RPMI 6666	> 5.00E+01	> 5.00E+01	> 5.00E+01	0.27
C15:0 (pentadecanoic acid)	Raji	> 5.00E+01	> 5.00E+01	> 5.00E+01	0.10
C15:0 (pentadecanoic acid)	SW1573	> 5.00E+01	> 5.00E+01	> 5.00E+01	0.33
C15:0 (pentadecanoic acid)	YAPC	> 5.00E+01	> 5.00E+01	> 5.00E+01	0.31
C15:0 (pentadecanoic acid)	NCI-H226	> 5.00E+01	> 5.00E+01	> 5.00E+01	0.77

Summary table for vehicle background

Cell line	Relative cell count (POC)			Doublings
	Mean	StdDev	CV	
JVM-2	100.00	4.01	0.04	4.04
Jiyoye	100.00	4.21	0.04	2.71
PANC-1	100.00	6.24	0.06	2.36
L-428	100.00	4.16	0.04	2.05
H9	100.00	6.89	0.07	2.68
HepG2	100.00	4.32	0.04	2.02
NCI-H69	100.00	4.70	0.05	1.26
JeKo-1	100.00	4.45	0.04	2.78
HT	100.00	6.61	0.07	2.02
T47D	100.00	3.02	0.03	1.72
TUR	100.00	4.36	0.04	4.29
SU.86.86	100.00	2.16	0.02	2.41
ZR-75-1	100.00	5.73	0.06	1.84
U-937	100.00	3.17	0.03	2.41
Ramos (RA 1)	100.00	4.53	0.05	3.25
SU-DHL-8	100.00	8.05	0.08	2.96
SR	100.00	5.85	0.06	3.62
SHP-77	100.00	6.40	0.06	1.92
BT474	100.00	5.71	0.06	1.26
CFPAC-1	100.00	8.65	0.09	2.66
A427	100.00	7.07	0.07	2.68
COR-L23	100.00	8.60	0.09	4.47
ChaGoK1	100.00	5.74	0.06	2.57
BT-549	100.00	7.04	0.07	2.38
NCI-H1573	100.00	7.83	0.08	1.32
NCI-H441	100.00	6.47	0.06	2.12
SW900	100.00	5.07	0.05	1.67
H69AR	100.00	3.28	0.03	2.16
DMS273	100.00	4.77	0.05	2.80
EFM-19	100.00	5.56	0.06	1.42
DOHH-2	100.00	8.75	0.09	3.47
NCI-H520	100.00	4.81	0.05	2.90
SKMES1	100.00	3.17	0.03	2.56
Capan-1	100.00	3.76	0.04	0.89
COR-L105	100.00	4.96	0.05	1.70
Daudi	100.00	3.90	0.04	2.59
DMS114	100.00	5.23	0.05	1.05
HuP-T4	100.00	4.90	0.05	3.88
HUH-6 Clone 5	100.00	7.09	0.07	2.54
MDA MB 231	100.00	5.79	0.06	2.36
KPL-1	100.00	14.73	0.15	2.71
NCI-H596	100.00	6.11	0.06	1.65
NCI-H460	100.00	7.01	0.07	4.60
HPAF-II	100.00	4.95	0.05	2.91
HLF	100.00	7.23	0.07	2.94
Raji	100.00	8.18	0.08	2.53
RPMI 6666	100.00	4.85	0.05	1.97
YAPC	100.00	10.57	0.11	2.66
SW1573	100.00	6.89	0.07	3.13
Mia PaCa-2	100.00	8.57	0.09	3.52

MDA MB 453	100.00	3.83	0.04	2.35
PSN-1	100.00	11.98	0.12	4.28
OCUG-1	100.00	5.09	0.05	3.49
NCI-H661	100.00	8.00	0.08	3.08
Hs 578T	100.00	4.95	0.05	1.76
A549	100.00	17.12	0.17	3.00
Hs 766T	100.00	3.07	0.03	2.00
MDA MB 415	100.00	4.86	0.05	1.36
NCI-H292	100.00	9.76	0.10	2.88
NCI-H446	100.00	10.75	0.11	1.29
MDA MB 436	100.00	3.26	0.03	1.25
Calu6	100.00	6.65	0.07	2.21
CA46	100.00	4.54	0.05	3.89
EB-3	100.00	7.34	0.07	2.53
DB	100.00	8.04	0.08	2.67
BT20	100.00	5.39	0.05	1.29
AsPC-1	100.00	6.60	0.07	1.15
Calu1	100.00	6.10	0.06	2.72
SK-BR-3	100.00	2.59	0.03	1.76
NCI-H226	100.00	22.94	0.23	1.84
